# Early Reduction of Microglia Activation by Irradiation in a Model of Chronic Glaucoma

**DOI:** 10.1371/journal.pone.0043602

**Published:** 2012-08-30

**Authors:** Alejandra Bosco, Samuel D. Crish, Michael R. Steele, Cesar O. Romero, Denise M. Inman, Philip J. Horner, David J. Calkins, Monica L. Vetter

**Affiliations:** 1 Department of Neurobiology and Anatomy, University of Utah, Salt Lake City, Utah, United States of America; 2 Department of Ophthalmology and Visual Sciences, Vanderbilt University, Nashville, Tennessee, United States of America; 3 Department of Neurosurgery, University of Washington, Seattle, Washington, United States of America; University of Regensburg, Germany

## Abstract

Glaucoma is a neurodegenerative disease that results in the progressive decline and ultimate death of retinal ganglion cells (RGCs). While multiple risk factors are associated with glaucoma, the mechanisms leading to onset and progression of the disease remain unknown. Molecular analysis in various glaucoma models has revealed involvement of non-neuronal cell populations, including astrocytes, Mueller glia and microglia, at early stages of glaucoma. High-dose irradiation was reported to have a significant long-term protective effect in the DBA/2J (D2) mouse model of glaucoma, although the cellular and molecular basis for this effect remains unclear. In particular, the acute effects of irradiation on specific cell populations, including non-neuronal cells, in the D2 retina and nerve have not been assessed. Here we report that irradiation induces transient reduction in proliferating microglia within the optic nerve head and glial lamina within the first week post-irradiation. This was accompanied by reduced microglial activation, with no effect on astrocyte gliosis in those regions. At later stages we confirm that early high-dose irradiation of the mouse head results in improvement of axonal structural integrity and anterograde transport function, without reduction of intraocular pressure. Thus reduced microglial activation induced by irradiation at early stages is associated with reduced optic nerve and retinal neurodegeneration in the D2 mouse model of glaucoma.

## Introduction

Glaucoma is a neurodegenerative disease that destroys vision through progressive degeneration of the optic nerve and decline and death of retinal ganglion cells (RGCs). This disease affects roughly 70 million people, and is a leading cause of non-curable vision loss [1]. Multiple risk factors contribute to glaucoma, including race, age and elevated intraocular pressure [2], [3]. However, how these factors influence neuronal loss in glaucoma is complex. In human patients, as well as in experimental animal models, there is evidence that age- and IOP-related RGC axon damage first occurs in the optic nerve head (ONH), where optic axons exit the eye [4]. But degenerative changes also affect other RGC compartments, localized to the retina, nerve and brain, in an asynchronous manner, impacting cell somata, dendrites and their synapses, myelinated axons, and axonal terminations [5], [6], [7].

Analysis of molecular and cellular changes in various animal models of glaucoma have suggested involvement of diverse non-neuronal cell populations, including astrocytes, Mueller glia and microglia, although the relative contributions of these glial cells to RGC degeneration or protection remain unclear [8]. Microglia are resident immune cells of the central nervous system that have been implicated in neurodegenerative diseases, including glaucoma [8], [9]. These glial cells respond to neuronal stress or injury with changes in distribution and cell activation, which involves simplification of cell morphology, expression of various growth factors and cytokines, and in some cases migration, proliferation or phagocytic activity [9], [10]. Notably, microglia reside in proximity to all RGC compartments impacted in glaucoma including dendrites and synapses, cell somata, and axons [11]. In human glaucoma, activated microglia have been found clustered in the region of the ONH, which is the site of initial axonal injury [12].

In the DBA/2J mouse (D2), which is an established model of inherited pigmentary glaucoma [13], microglia become activated prior to evidence of RGC structural decline [11]. Spatially, activated microglia first localize to the ONH and lamina around unmyelinated optic axons [11], the presumed sites of initiation of optic neuropathy in D2 mice [14], [15]. At later stages microglia activation spreads to the peripheral inner retina [11], and this is associated with slow microglial proliferation that doubles retinal microglia numbers from 4 to 10 months of age [16]. Consistent with involvement of microglia in glaucoma, microarray analysis of the D2 mouse has shown that there are changes in gene expression within the retina as well as the ONH that suggest an innate immune response [17], [18], [19]. Similar changes in gene expression have been reported in microarray studies of the retina following acute IOP elevation in various species [20], [21], [22], [23]. Furthermore, early and chronic deactivation of microglia by administration of minocycline results in improved RGC axonal integrity and transport function [24]. Thus microglia activation represents a significant and early component of pathology in glaucoma.

High-dose radiation treatment of the entire animal with syngeneic bone marrow transfer (BMT) had an unexpected and remarkably profound protective effect on glaucomatous damage in the D2 mouse [25]. Neither iris disease nor IOP elevation were affected by treatment, but there was dramatic improvement in axon preservation out to 14 months of age, when most D2 mice normally show severe optic nerve pathology. In addition, there was protection against RGC somal loss and excavation of the ONH. The protective effect was also observed with irradiation of the head or eye without BMT [26]. In these studies irradiation was performed from 5 to 8 weeks of age, which is just prior to significant microglial activation in the D2 mouse [11]. Thus, it is possible that treatment had an effect on microglia with the potential to impact the subsequent course of degeneration.

Here we tested the effects of high-dose irradiation of the head region alone on D2 mice and found transient reduction in proliferating microglia within the ONH and glial lamina, with maximal effect within the first week post-irradiation. This was accompanied by reduced microglial activation within the central retina and proximal optic nerve compartments, with no effect on astrocyte gliosis in those regions. We also found significant improvement in axonal survival and anterograde transport without reduction of IOP, confirming that bone marrow transfer is not required to abate degeneration. Thus, one consequence of high-dose irradiation is to acutely reduce the levels of microglia activation in this glaucoma model.

## Materials and Methods

### Ethics Statement

This study was performed in strict accordance with the recommendations in the Guide for the Care and Use of Laboratory Animals of the National Institutes of Health. The protocol was approved by the Institutional Animal Care and Use Committee of the University of Utah (Animal Welfare Assurance Number: A3031-01). All procedures were performed under avertin anesthesia, and every effort was made to minimize suffering.

### Animals

Adult DBA/2J mice (Jackson Laboratory (JAX), Bar Harbor, ME; stock # 000671) served as an experimental model of inherited pigmentary glaucoma, and its DBA/2J-Gpnmb+/Sj substrain (stock # 007048) as non-glaucoma control [27], [28]. These strains are referred to as D2 and D2G mice, respectively. Mice were bred and aged at the University of Utah in a pathogen-free barrier facility under a 12 hr light/dark cycle, and every 4 generations breeders purchased from JAX were introduced to avoid genetic drift. Mice under anesthesia were identified with ear tattoos (Harvard Apparatus, Holliston, MA). D2.CX3CR1^gfp/+^ mice, where microglia (and other myeloid cells) specifically express GFP under the control of fractalkine receptor locus [29], were generated after backcrossing B6.129P-*CX3CR1^tm1Litt^*/J mice (JAX stock #005582) onto the D2 background for over 10 generations. All animal maintenance and procedures were approved by the University of Utah Institutional Animal Care and Use Committee, and followed the Statement for the Use of Animals in Ophthalmic and Vision Research from the Association for Research in Vision and Ophthalmology (ARVO).

### Head irradiation

Four to six week-old female and male D2 mice had the rostral half of their heads exposed to a single high dose of electromagnetic radiation (8 Gy or 800 rads, 1 Gy/min) delivered by a biological X-ray generator (X-Rad 320, Precision X-Ray, North Branford, CT). To restrict irradiation to the eyes and visual pathways, each mouse was positioned inside a 50 mL conical tube with its muzzle exposed through the truncated end of the tube, while the rest of the body was shielded with two 6 mm-thick lead plates. Irradiation targeted an area delimited by the base of the ears and the beginning of the eyes, sparing the nose. The head region and the depths targeted were maintained constant across animals with the aid of reference collimated light and focusing LEDs. During the procedure, mice were anesthetized via intraperitoneal Avertin injection, and kept warm over isothermal pads (Braintree Scientific, Braintree, MA).

### Intraocular pressure (IOP) measurement

IOP was measured monthly from 2 to 8 months of age in both irradiated and untreated D2 mice using the TonoLab (Colonial Medical Supply, Franconia, NH), as previously described [11]. Each data point represents individual (per eye) monthly, mean IOP values (20 readings). Mean value per age and experimental group were tabulated, as well as sample size.

### Optic nerve histology and analysis

Axon counts were obtained in optic nerve cross-sections (2 µm-thick) from 9–12 month-old irradiated and control D2 mice. Optic nerves 2 mm proximal to the globe were stained with paraphenylenediamine (PPD) and counterstained with toluidine blue then prepared for plastic embedding and semithin sectioning, as previously described [30], [31], [32]. Using differential interference contrast optics at 100× magnification, cross-sections of optic nerve were photographed en montage using an Olympus Provis AX70 microscope. Custom algorithms were used to identify and count axons across the entire cross-section, as previously described [33].

### Anterograde transport analysis

To measure the preservation of RGC axonal transport from the retina to the superior colliculus (SC), cholera toxin β-subunit (CTB) transport was assayed in 10–12 month-old irradiated and non-irradiated mice as recently described [31]. Briefly, 1 µL of 1% CTB conjugated to Alexa Fluor 488 nm (Molecular Probes, Eugene, OR) in sterile PBS was injected intravitreally, using a 33 G needle attached to a 25 µL Hamilton syringe (Hamilton Co., Reno, NV). After 2 days, serial coronal brain sections (50 µm) were collected from mice prepared by transcardial perfusion with 4% paraformaldehyde in PBS. CTB signal was imaged throughout alternate SC sections to reconstruct the total retinotopic map in the superficial layers. CTB signal was normalized to the background of the deeper, non-retinorecipient layers and label and density was calculated and plotted across the total SC. CTB density is represented colorimetrically, and the fraction of intact transport map is defined as the percent area with CTB signal ≥70%.

### Retrograde Transport Analysis

To measure retrograde transport of cargo from superior colliculus to retina within the retinal ganglion cell axons, a small burr hole was drilled through the skull above the superior colliculus in anesthetized mice; FluoroGold was injected (1 µL, bilaterally) as described (Buckingham, et al., 2008). The holes were plugged with Gelfoam (Pfizer) soaked in 5% FluoroGold. Three days later, retinas were collected from mice after transcardial perfusion with 0.1M PBS followed by 4% paraformaldehyde in PBS, dissected out, immunolabeled with NeuN (1∶500, Millipore, Temecula, CA) and FluoroGold (1∶500, Millipore), mounted flat with gelvitol, then coverslipped. Unbiased stereological analysis of NeuN and FG-positive cell number was undertaken in wholemount retinas using the optical fractionator module within StereoInvestigator software (MicroBrightfield, Middlebury, VT).

### Retinal immunofluorescence, microscopy and analysis

Processing, immunostaining and imaging of retinal wholemounts and cryosections (30 µm-thick) including the ONH, OL and proximal ON regions were performed as previously described [11]. We used polyclonal antibodies against Brn3 to identify RGCs, which recognize its three isoforms, (1∶50, C-13; Santa Cruz, Santa Cruz, CA), Iba1 to identify microglia (1∶1,000; Wako, Richmond, VA), and monoclonal antibodies against GFAP to label astrocytes (1∶1,000; Sigma, St. Louis, MO), and against the 68 and 200 kDa isoforms of phosphorylated neurofilament to identify optic axons (1∶50, clone 2f11; Dako, Carpinteria, CA). An antibody recognizing PCNA was used to identify mitotic cells (1∶500, clone PC10; Dako, Carpinteria, CA), which required prior antigen unmasking with a solution of 0.18 mM citric acid, 77 µM sodium citrate, pH 6 for 15 min at 90°C. Immunostaining of retinal wholemounts and sections consisted of a 3-day incubation of three different primary antibodies at 4°C, followed by 2 hr incubation in Alexa Fluor-conjugated secondary antibodies (1∶400, Molecular Probes). Confocal images were acquired with identical settings for each marker to allow comparison across samples (A1 and NIS-Elements 3.1, Nikon, Melville, NY), and are published with minimal adjustment of brightness (Photoshop CS3, Adobe Systems, San Jose, CA). Each flat mounted retina was imaged by acquiring 625 XY images throughout inner 25–35 µm of the retina (0.7 µm-step). For analysis, these images were stitched to produce a high-resolution image (0.41 µm/pixel) of the retinal surface, and published as maximal intensity projections that span the nerve fiber layer (NFL) and ganglion cell layer (GCL). To represent variations in relative intensity of protein levels, some published images were pseudocolored as indicated by a color code bar. Confocal images of cryosections of the central retina and proximal optic nerve regions show 20-µm thick maximal intensity projections.

### Microglia cell counting

Total and proliferating microglial cells were counted in images of radial sections along the central retina and proximal optic nerve from irradiated D2 mice (1, 7 and 30 days post-treatment) and aged-matched untreated D2 mice. Cells were counted within areas localized to the head, laminar and myelinated optic nerve regions, defined as ONH (150 µm below NFL-plane), OL (100 µm from choroidal plane), and ON (250 µm from OL). Briefly, total microglia numbers were obtained from counting Iba1-positive cells with an identifiable Hoechst-stained nucleus (total microglia) within each nerve region. Proliferating microglia were recognized as cells with cytosolic Iba1 and nuclear PCNA, unambiguously identified by tracing their linear density profile across the cell soma, in single optical slice views. Cell counts in each compartment were normalized to represent percentage of proliferating cells and cells per mm^2^.

### Detection of apoptotic microglia

To detect apoptosis in microglial cells, avoiding artifacts derived from euthanasia and histology, we used D2.CX3CR1^gfp/+^ mice, in which microglia express GFP, and delivered SR-FLIVO, a red-fluorescent tracer that selectively forms covalent bonds with active caspases *in vivo* (Immunochemistry Technologies LLC, Bloomington, MN). Following manufacturer's protocol, 100 µL FLIVO was administered to 2–3 month-old mice via tail vein injection 8 hr after irradiation and 8 hr before sacrifice, which was replicated in non-irradiated control mice. Retinas were dissected and flat mounted fresh, then fixed for 30 min and counterstained with Hoechst 33258 (Invitrogen, Carlsbad, CA). Confocal images were collected for each entire retina as described above.

### Quantitative real time PCR analysis

Levels of Iba1 mRNA expression were measured in a sample that included the central 1-mm of retina, with its ONH and OL, using techniques and primers previously described [11]. Iba1 expression was assessed 1 week and 4 to 6 weeks after irradiation, and compared to untreated age-matched D2 and D2G samples (n = 10 per timepoint and strain). Data is presented as mean fold change normalized to β-actin expression within each sample.

### Statistical analysis

Statistical significance was determined using two-way analysis of variance, followed by two-tailed Student's t-test (GraphPad, La Jolla, CA). Statistical significance is indicated by asterisks, p<0.05 (*) and p<0.01 (**). Sample sizes are also reported in Figure legends.

## Results

### High dose head irradiation alters early microgliosis

**Figure 1:**
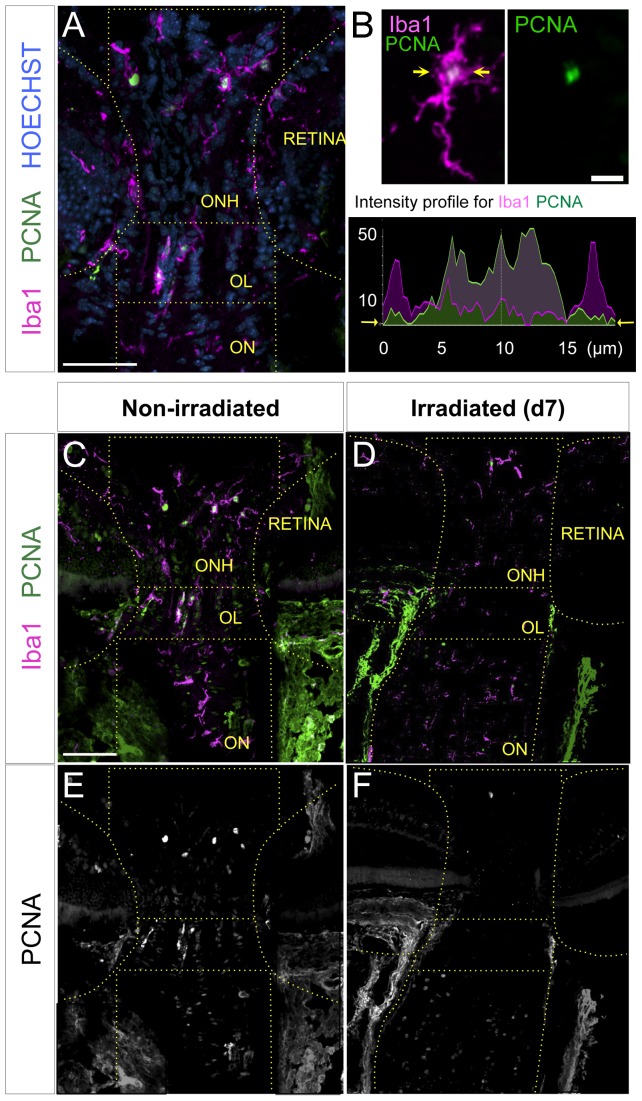
Irradiation results in depletion of proliferative microglia within the proximal optic nerve regions. *(A)* Iba1-positive cells with and without nuclear expression of the proliferative marker (PCNA) were detected in radial sections along the ONH, OL and proximal ON (delineated) shown in a single 1 µm optical slice. *(B)* Overlay of Iba1 and PCNA, plus single-channel view for PCNA. Scale bar represents 10 µm. The colocalization of Iba1 and PCNA to individual cells was confirmed by tracing their linear intensity profiles in single optical slices (between the arrows). Cycling microglial cells were positively identified by the nuclear localization of PCNA expression within the cytoplasmic Iba1 expression (graph). *(C, E)* Non-irradiated D2 mice show concentration of proliferating microglia within the unmyelinated ONH and OL, shown in a maximal Z-projection. Cycling microglia have a green or white nucleus in the overlay view of Iba1 and PCNA stainings, which is shown as single PCNA-channel for clarity. *(D, F)* 7 days (d7) after irradiation microglia proliferation appears negligible along the proximal nerve, as shown in a maximal Z-projection. Scale bars, 100 µm.

One effect of high-dose irradiation is to kill dividing cells, so we hypothesized that irradiation may be affecting a proliferative glial cell population in the retina, optic nerve head (ONH), optic lamina (OL), or optic nerve (ON). Previous analysis has shown that microglia represent the only significant dividing cell population in the D2 retina, doubling their numbers from 4 to 10 months of age [16]. Furthermore, we previously showed that microglial cell density increased in the central inner retina and ONH in D2 mice by 3 months of age versus D2G controls, suggesting increased proliferation [11]. To directly assess whether microglia are proliferating at 4 to 6 weeks of age (the time of irradiation) in the central retina, ONH, OL and/or proximal ON, radial retinal sections were double-immunostained for Iba1 and PCNA to identify and quantify dividing microglial cells within each region (Fig. 1A, B). Numerous PCNA+/Iba1+ cells were detected in the ONH, OL and ON from non-irradiated D2 animals, suggesting the presence of proliferating microglia (Fig. 1C, E). Few PCNA+/Iba1+ cells localized to the peripheral retina (data not shown), consistent with previous analysis [16].

To assess the effects of irradiation on microglia in the eye, we selected mice from 4 to 6 weeks of age, shielded the body with lead plates, and delivered a single high dose of X-irradiation (8 Gy) to the rostral half of the head spanning the eye region and visual pathway (see Methods and Fig. S1). IOP was measured monthly from 2 to 8 months of age in both non-irradiated and irradiated mice. The irradiated cohort of D2 mice still showed the expected IOP elevation (>20 mm Hg) by 7–8 months (Fig. S2), consistent with previous reports [25], [26]. We then assessed the effects of irradiation on proliferating microglia and found that in irradiated D2 mice, the presence of PCNA+/Iba1+ cells was dramatically reduced one week post-irradiation (Fig. 1D, F). To determine whether this was due to induction of apoptosis we delivered SR-FLIVO probe to 2–3 month-old D2.CX3CR1^gfp/+^ mice by tail vein injection 8 hours post-irradiation, then sacrificed animals 8 hours later for retina dissection and analysis. We detected apoptotic microglia in irradiated mice, which were mostly concentrated in the central retina, while apoptotic microglia were absent in non-irradiated controls (Fig. S3).

**Figure 2:**
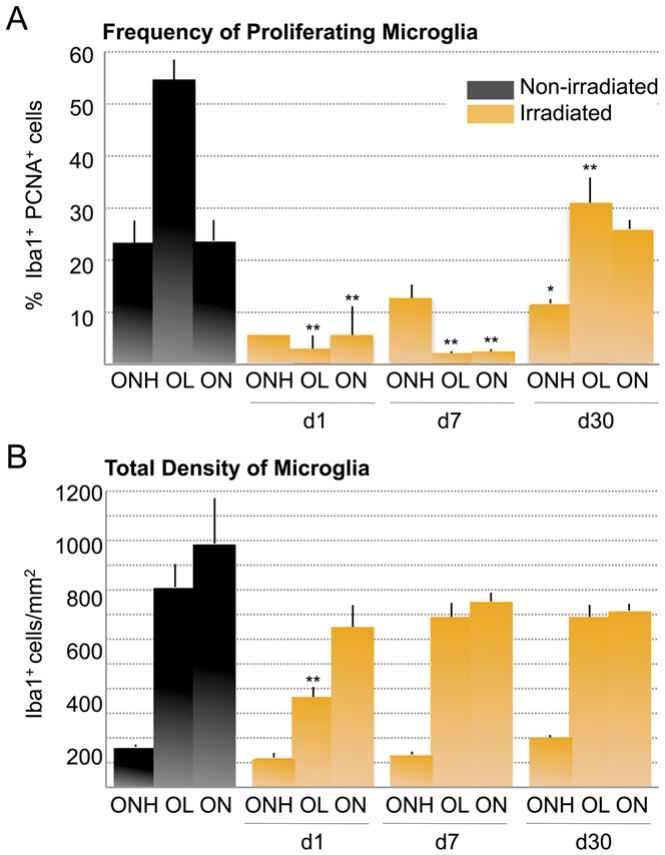
Irradiation reduces microgliosis in the unmyelinated optic nerve. Quantification of the proportion of cycling microglial cells (PCNA and Iba1-positive) per optic nerve compartment in non-irradiated and irradiated D2 mice (n = 6 samples per timepoint and experimental group). The statistical significance indicated for each compartment and timepoint is relative to non-irradiated controls. *(A)* In non-irradiated D2 mice aged 4 to 6 weeks, the proportion of proliferating microglia is ∼50% within the OL, and ∼25% within the ONH and ON. At 1 day and 7 days after irradiation, numbers of cycling microglia are reduced in all optic nerve portions, with maximal reduction within the OL (p<0.001), and with significant effect in the ONH and proximal ON (p<0.01). 30 days post-treatment the unmyelinated ONH and OL compartments have recovered only half of its original proliferating microglia, whereas the proximal ON has been repopulated. *(B)* Total number of Iba1-positive microglia (per area) within each optic nerve compartment. The density of total microglia demonstrates that irradiation has an acute and specific effect on cycling microglia resident within the OL (p<0.01).

To quantify the effect of radiation on proliferating microglia, we counted the percent of Iba1+ PCNA+ microglia localized to the ONH, OL and proximal ON in untreated D2 animals, and at 1, 7 and 30 days post-irradiation. In non-irradiated 4 to 6 week-old D2 mice we found proliferating microglia in all three regions, but detect the highest proportion in the lamina (OL), representing half of the microglia population in this region (Fig. 2A). At both 1 and 7 days post-irradiation, proliferating microglia were dramatically reduced in the ONH, OL and ON, with some recovery in the ONH by 7 days. By 30 days post-irradiation the proportion of cycling microglia within both the ONH and OL had recovered to nearly half of their original numbers, while the repopulation within the first 250 µm of ON was complete (Fig. 2A). When we assessed changes in overall density of microglia, we found a general reduction in all compartments at 1 day post-irradiation, but most significantly in the OL, consistent with the high proportion of proliferating microglia in that region (Fig. 2B). Microglial cell density was restored to levels approaching non-irradiated D2 mice by 7 days post-irradiation and was maintained out through 30 days. Thus, we conclude that irradiation has an acute and significant effect by reducing the presence of proliferating microglia.

### Irradiation results in reduced levels of microglia activation

**Figure 3:**
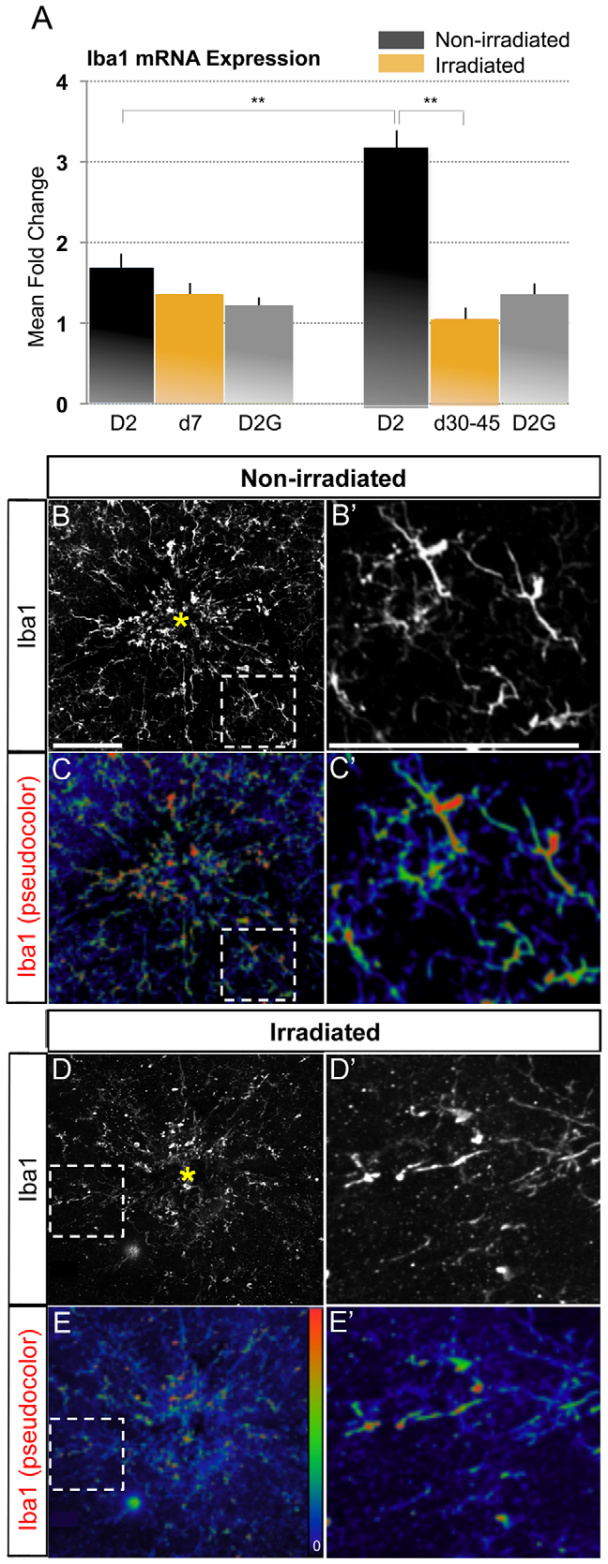
Irradiation reduced early microglia activation within the central retina and proximal optic nerve compartments. *(A)* Quantitative RT-PCR analysis of Iba1 transcript in a sample including the central 100 µm of retina, ONH, OL and proximal 200 µm of myelinated optic nerve, collected from non-irradiated D2 and non-glaucoma D2G, and irradiated D2 mice (n = 10 per group and age). 7 days post-irradiation there are comparable levels of Iba1 expression as in age-matched non-irradiated D2 or D2G samples. 30 to 45 days after irradiation Iba1 expression is significantly lower than the elevated levels of expression seen in non-irradiated D2 tissue at this age (p<0.01), and is comparable to levels of Iba1 expression in age-matched non-glaucoma D2G mice. *(B, C)* Representative retinal wholemounts from non-irradiated and irradiated D2 mice at 3 months of age show clustering of large, activated microglia with high Iba1 expression within the central retina and optic disc (yellow asterisk) in non-irradiated conditions. In age-matched irradiated mice few high-Iba1 expressing microglia are detectable, and display little clustering. *(D, E)* In non-irradiated D2 retinas, Iba1 expression (pseudocolor code indicated as inset bar) is highly upregulated in the microglial cells concentrated at the optic disc, while only few cells show high expression levels in untreated D2. (B'–E') High magnification views of framed areas shown in B–E. Scale bars, 100 µm.

Since the numbers of proliferating microglia were acutely reduced by high dose irradiation, we sought to investigate whether irradiation also had an acute effect on the level of microglial activation, as well as an impact on the peak activation detectable by 3 months of age in the central retina and ONH of D2 mice [11]. To quantify changes in Iba1 mRNA expression 7 days and 30 to 45 days post-irradiation, we performed quantitative RT-PCR on mRNA isolated from microdissected samples that included the central 100 µm of central retina, the unmyelinated optic nerve regions and the first ∼200 µm of myelinated optic nerve. We compared these samples to age-matched control samples from non-irradiated D2 and D2G retinas. After 7 days, irradiation did not alter the levels of Iba1 expression, which are low at this age and were comparable between the three groups analyzed. Normally there is an increase in microglia activation in the central retina and proximal nerve region by 3 months of age [11], which is also observed here as indicated by an elevation of Iba1 expression (Fig. 3A). In irradiated D2 tissue at this age, which is 30 to 45 days after irradiation, we found a significant reduction in Iba1 expression down to levels corresponding to those in tissue from non-glaucoma D2G mice (Fig. 3A), suggesting that irradiation prevents the increase in microglia activation. We conclude that, after high dose irradiation, the microglia localized to the central retina and proximal nerve region show sustained reduced levels of activation, comparable to non-glaucoma controls.

The striking finding that irradiation lowers the levels of Iba1 mRNA expression to control levels by 3 months of age was confirmed by immunostaining retinal wholemounts from non-irradiated and irradiated D2 mice for Iba1. To assess microglia activation, microglia localized to the central retina and optic disc were imaged at high resolution. We found that non-irradiated D2 retinas show clustering of microglia with large soma size and reduced branching within the central retina, demonstrating cell activation (Fig. 3B, B'). In contrast, irradiated retinas show microglia with small somata and thinner branches, and therefore with reduced levels of activation (Fig. 3D, D'). High levels of Iba1 expression was observed in numerous microglial cells in non-irradiated D2 retinas (Fig. 3C, C'; pseudocolored, with red representing the highest levels of expression), and this is clearly reduced in age-matched tissue after irradiation (Fig. 3D, E; pseudocolored). Our findings indicate that irradiation suppresses the intense microglia activation, characteristic of the early D2 retina.

### Irradiation did not induce early gliosis

Glaucoma-like pathology in the D2 retina is also associated with significant astrocyte and Mueller cell reactive gliosis, which is evident by upregulation of GFAP expression [16]. To assess whether irradiation induces gliosis at 3 months of age, we generated radial sections through the central retina and its proximal optic nerve regions and immunostained them for GFAP. Non-irradiated and irradiated D2 mice showed similar levels and patterns of GFAP expression, confined in the retina to the NFL where astrocytes reside, and spanning the proximal optic nerve segments (Fig. S4).

### Irradiation reduces RGC axon degeneration

**Figure 4:**
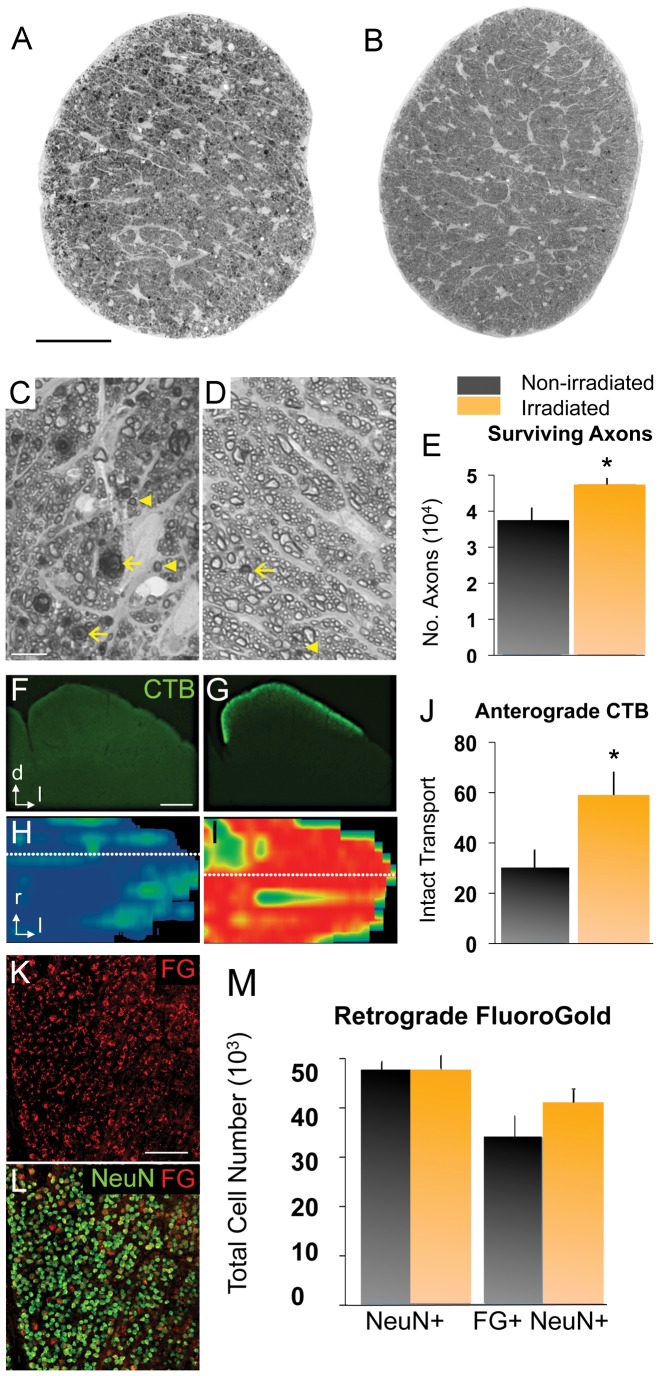
Head irradiation protects D2 mice from optic nerve degeneration. *(A, B)* Optic nerve cross-sections stained with PPD, from naïve (11 mo old) and irradiated (12 mo old) D2 mice. Scale bar, 250 µm. (C, D) Higher magnification view of the same nerves. Scale bar, 10 µm. *(C)* Non-irradiated D2 nerves show abundant degenerating axon profiles with dark, condensed axoplasm (arrowheads) and disorganized myelin sheaths (arrows), as well as extensive gliosis. *(D)* Irradiated D2 nerves mostly show healthy axons with clear axoplasm and uniform myelin sheaths, and rare degenerating profiles (arrows). *(E)* Total optic axon counts from irradiated D2 nerves (n = 29; 12 mo old) show a significant increase in mean axon number compared to non-irradiated D2 mice (n = 16; 10 and 11 mo old; p<0.01). *(F–J)* The anterograde transport of CTB by RGC axons from the retina to the superior colliculus (SC) was measured in non-irradiated (n = 16; 10 and 11 mo old) and irradiated D2 mice (n = 17; 12 mo old). *(F)* Non-irradiated D2 mice (11 mo old) showed severe loss of CTB signal, as seen in coronal section of the SC (dorsal, d; lateral, l). Scale bar, 500 µm. *(G)* Irradiated D2 mice (12 mo old) showed persistent CTB transport to the superficial layers of the SC. *(H, I)* Reconstruction of the retinotopic SC map, showing the density of CTB signal (red, green and blue indicate 100, 50 and 0% density, respectively). Dotted lines indicate location of sections shown in F and G; rostral, r; lateral, l. *(H)* Non-irradiated D2 mice showed near total CTB depletion. *(I)* Irradiated D2 mice showed almost complete CTB label and a normal retinotopic pattern. *(J)* Irradiation significantly protected axonal anterograde transport in D2 optic nerves (p<0.05). K) Flat-mount of an irradiated D2 retina at 9 mo of age showing FG labeled cells (red). L) Most NeuN+ RGCs (green) in this retina are retrogradely labeled with FG (red). Scale bar, 100 µm. M) Quantitative stereology of both NeuN+ RGCs and FG+/NeuN+ RGCs in flat-mounted retinas. Non-irradiated retinas (dark bars, n = 8; 9–10 mo old) and irradiated retinas (yellow bars, n = 8; 9–10 mo old) showed no differences in the total number of NeuN+ RGCs per retina between treatments, but slightly more retrogradely labeled FG+ RGCs in the irradiated retinas. Error bars = SEM.

After 9 months of age, optic nerves from non-irradiated D2 mice showed significant structural degeneration, including an abundance of dystrophic axons with expanded diameters and compacted myelin sheaths, dense and dark axonal profiles with multilayered myelin sheaths, and enlarged glial cell somata, indicative of gliosis (Fig. 4A, C), consistent with previous reports [32]. In contrast, irradiated mice showed relatively normal optic nerves with few degenerating axonal profiles and generally intact myelin sheaths (Fig. 4B, D). Since D2 mice typically show significant axon loss by 10 to 12 months of age we quantified axons to assess whether irradiation had a protective effect. We found that irradiated D2 mice had significantly larger axon numbers as compared to untreated D2 mice, indicating reduction of RGC axonal degeneration (Fig. 4E).

Another feature of axonal decline in the D2 mouse is progressive loss of anterograde axonal transport from the retina to the superior colliculus (SC), which is severely compromised by 11–12 months of age [31]. To assess whether this is improved by irradiation, we performed intravitreal injection of cholera toxin β-subunit (CTB) in live mice, which is actively taken up by RGCs and transported by their axons to the superficial layers of the SC. Coronal sections of 11-month old SC of non-irradiated D2 mice reveal almost complete loss of CTB label along the medial-lateral extent of the SC, throughout its entire length (Fig. 4F, H). In contrast, irradiated 12-month old mice showed persistent CTB label throughout the entire SC (Fig. 4G, I). We quantified this effect across multiple animals by calculating the percentage of animals with intact anterograde axonal transport, as defined by ≥70% CTB signal density, and found a significant increase in irradiated D2 mice compared to untreated animals (Fig. 4J). Thus, axonal transport function is also robustly preserved in D2 mice by high-dose head irradiation.

Previous reports have demonstrated significant decline in retrograde transport of FluoroGold tracer in advance of significant RGC death (Buckingham, et al., 2008). Significantly, irradiation did not have a direct toxic effect on cells in the ganglion cell layer since the NeuN+ cell numbers did not differ between irradiated and non-irradiated control (Fig. 4M). Irradiated mice injected with FluoroGold demonstrated no significant improvement of retrograde transport of the tracer as compared to non-irradiated mice, although there was a trend towards increased transport (Fig. 4M).

#### Irradiation improves retinal ganglion cell integrity

**Figure 5:**
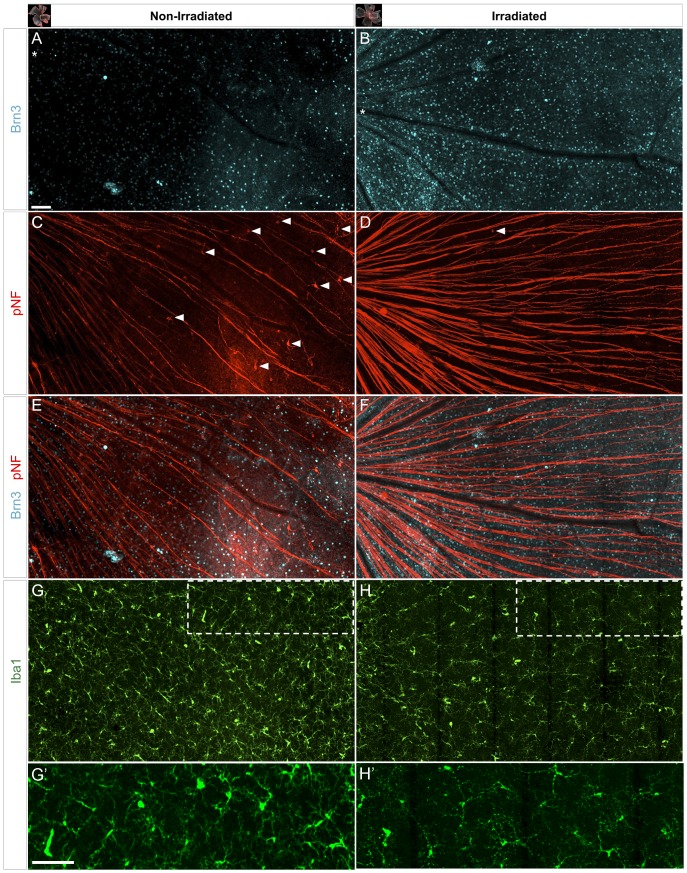
Irradiation reduces RGC decline and microglia activation within the retina. Confocal images of triple-immunostained (Brn3, pNF and Iba1) flat-mounted retinas from non-irradiated (11 mo old) and irradiated (12 mo old) D2 mice (n = 8 retinas per group). Representative fields (∼1.6×0.9 mm^2^) show matching position (optic disc at left of asterisks) and a projection of the NFL and GCL, from central to peripheral eccentricities. *(A–B)* Most of the non-irradiated retina displays low numbers of RGCs expressing Brn3, whereas irradiated mice maintain a higher density of brightly labeled RGCs. *(C, D)* Co-immunostaining for pNF in the same non-irradiated retina shows a reduced number of optic axons, thinner axonal bundles and defasciculated axons that show beaded profiles in their entire length. In the irradiated retina, RGC axons are organized in bundles that show a higher and uniform pNF content, and some fragmentation mostly limited to the peripheral area. Only non-irradiated retinas show abundant RGCs with somadendritic accumulation of pNF (arrowheads). *(E, F)* Overlay of Brn3 and pNF staining reveals the colocalization of declining axons and sparse Brn3-positive RGCs in the non-irradiated D2 retina, in contrast to healthier, fasciculated axons in the irradiated mice. *(G, H)* Reflecting the abnormalities shown by the RGC markers, microglia (Iba1) had altered tiling around RGC axons and variable, increased density in non-irradiated retinas. Activated microglia, recognizable by their enlarged somata and reduced branching, were abundant in the non-irradiated retina, and infrequent in the irradiated ones. (G', H') High magnification views of framed areas shown in G, H. Scale bars, 100 µm.

Since RGC optic axon structure and transport function were preserved by irradiation, we investigated whether there is also preservation of intraretinal RGC compartments. To identify and assess the health status of RGCs, we collected retinas from 12 month-old mice that had received high-dose head irradiation at 4 to 6 weeks of age and, and compared these to non-irradiated D2 controls. We performed immunostaining in whole retinas with a Brn3 antibody that recognizes all isoforms of Brn3, a POU homeodomain transcription factor that identifies the majority of adult mouse RGCs. Brn3 expression is known to downregulate in aged D2 retinas prior to RGC loss (Buckingham et al., 2008; Soto et al., 2008). Confocal images of retinal flat-mounts showed that irradiated mice displayed a higher density of RGC somata expressing Brn3 compared to non-irradiated mice, as well as relatively more intense levels of Brn3 expression (Fig. 5A, B). Half of the non-irradiated D2 retinas analyzed (4/8 retinas) showed almost complete or severe loss of Brn3 immunostaining throughout the entire GCL, while only one irradiated retina (1/8 retinas) showed a significant portion of the retina with reduced Brn3, with none showing the most severe loss.

In the same tissue we performed co-immunostaining for phosphorylated neurofilament (pNF), which is normally localized to optic axons, but aberrantly accumulates within the somata, dendrites and intraretinal axon segment of declining RGCs in D2 mice [15]. We found that in non-irradiated D2 retinas, there were reduced numbers of pNF-labeled axons overall, as well as beading or fragmentation of the remaining axons (Fig. 5C). In contrast, RGCs in irradiated mice showed uniform levels of pNF expression along their intraretinal axons, although axonal beading was present in the far peripheral retina (Fig. 5D). Somal and dendrite accumulation of pNF immunostaining was detected in most non-irradiated D2 retinas, particularly in RGCs localized to the mid- and far-peripheral retina (Fig. 5C, arrowheads), and in some cases somal pNF was observed within every declining RGC in the far-periphery (4/8 retinas). However, irradiated retinas had very few RGCs with somal pNF build up, with only low levels of accumulation (Fig. 5D, arrowhead; 7/8 retinas). The merged view of the Brn3 and pNF immunostainings reveals that in the non-irradiated retina there is colocalization of dystrophic axons to areas with reduced density of Brn3-positive cells, while these areas are reduced, but not completely absent, in retinas from irradiated mice (Fig. 5E, F).

Since microglia activation is associated with neuron decline and degeneration in glaucoma and other neurodegenerative conditions, we immunostained the same tissue for Iba1 to assess the distribution and presence of activated microglia, identifiable by enlarged somata, thick processes and upregulation of Iba1 expression. We observed higher density of Iba1-positive microglia in non-irradiated D2 retinas (Fig. 5G), in comparison to irradiated ones (Fig. 5H). Moreover, non-irradiated retinas showed a more irregular tiling of microglia resident in the NFL and GCL (Fig. 5G, H). The presence of activated microglia was noticeably reduced in irradiated retinas, presumably due to reduced RGC decline and degeneration. Most cells showed complex, slender processes and small somata (Fig. 5H, H'), versus the conspicuous soma and processes of activated microglia in non-irradiated retinas (Fig. 5G, G'). Thus, head irradiation results in overall improvement of RGC integrity at 12 months of age, when the RGC somal loss by apoptosis is quite apparent in a majority of D2 mice [13], [34], [35], [36]. The late effect includes preservation of Brn3 expression and reduction of pNF buildup, in conjunction with reduced microglia activation.

## Discussion

Here we examined the acute effects of high-dose irradiation on microglia in the D2 mouse glaucoma model. We found that irradiation causes an immediate reduction in proliferating microglia in the optic nerve head and glial lamina region, as well as a sustained reduction in the levels of microglia activation in the central retina, optic nerve head and laminar region. We also confirmed that irradiation of the head alone reduces RGC degeneration, improving both the structural integrity of the axons as well as anterograde axonal transport at later stages. While the effects of high dose irradiation on neuronal tissue are complex, these findings suggest that a component of the response to irradiation involves changes in microglia.

High-dose irradiation has previously been shown to have significant effects on microglia, particularly in the young brain where proliferating microglia are more abundant than in the adult. For example, following high-dose irradiation of rats at P9 or P21, apoptotic microglia were evident by six hours, with a significant decrease in microglia cell numbers seven days later [37]. In the D2 retina, it has previously been shown that the major proliferating cell population is microglia [16], consistent with our observation that these cells are vulnerable to irradiation. Previously we reported microgliosis, as well as clustering and activation of microglial cells in the inner central retina and unmyelinated optic nerve regions (ONH and OL) in D2 mice at early ages [11]. In this study we found evidence for proliferating microglia concentrated in the OL by 1 mo of age, which were depleted by high-dose irradiation. In some cases, whole brain irradiation has been associated with increased microglia activation coupled with secondary astrocyte gliosis [38], although this was not observed here. In the D2 retina, the effect may target proliferating microglia, which are selectively increased in this disease model.

In this study, irradiation was performed in young D2 animals, from 4 to 6 weeks of age, which is prior to the onset of detectable neurodegeneration of RGCs. Since microglia activation can be detrimental to neurons [9], it is possible that the protective effect of irradiation is due to selective depletion of an activated, proliferating population of microglia, resulting in less neuronal damage. In support of this idea we have found that other interventions that reduce the levels of microglia activation, such as long-term minocycline treatment, can improve RGC axon integrity and axon transport function [24]. High dose irradiation with bone marrow transfer has also been shown to be neuroprotective in another neurodegenerative disease, Sandhoff's disease, which is an inherited lysosomal storage disorder causing acute and rapid neuronal decline usually leading to early childhood death [39]. Interestingly, neurodegeneration in this disease is preceded by extensive microglia activation in both human cases as well as in a mouse model of the disease, which was prevented by the irradiation and bone marrow transfer procedure in mice [39]. In this case, the effects of irradiation versus bone marrow transplantation were not tested. Nevertheless, it raises the possibility that irradiation may have similar neuroprotective effects in other disease models since early microglial activation has been reported for a number of neurodegenerative diseases [40]. However caution is warranted, since radiation retinopathy associated with adverse effects on retinal vasculature can be a significant complication of irradiating the retina, with few therapeutic options [41], [42], [43].

It is also possible that irradiation has a direct effect on other retinal cell populations, since there is precedence for irradiation impacting neural tissue. For example, therapeutic irradiation of the brain is used for treating brain tumors, but is known to induce cognitive deficits [44], [45]. Some of this may be due to the depletion of dividing neural stem cells and neural precursors in the ventricular zone of some brain regions [44]. This is unlikely to be a factor in this study since there are no dividing neural precursors or stem cells within the retina at this stage, with only microglia and pericytes dividing in the D2 retina [16]. In addition, postmitotic neurons are generally thought to be more radio-resistant [46]. We did not observe any significant acute effects of irradiation on RGC structural integrity (data not shown), suggesting that RGCs are relatively resistant to radiation-induced damage.

More subtle effects on neurons have been associated with irradiation. For example in hippocampal pyramidal neurons irradiation caused a loss of expression of some neuronal genes, such as NeuN, within 7 days [47]. In addition, irradiation has been shown to affect behaviorally induced expression of the immediate-early gene *Arc* (activity-regulated cytoskeleton-associated protein), suggesting alteration of neuronal function [48]. However, we observed normal expression of Brn3 and NeuN, suggesting that RGC gene expression is not negatively affected by irradiation. While these other studies suggest that irradiation causes reduction of neuronal integrity and function, it remains possible that exposure of the retina to high-dose irradiation alters neuronal gene expression, potentially rendering RGCs resistant to subsequent insult in this glaucoma model.

Retinal pericytes, which are associated with the retinal vasculature, also proliferate in the D2 retina [16], so these cells may also be impacted by high-dose irradiation, as suggested by our detection of activated caspases in diminutive cells along vessel walls. In fact, in other studies irradiation has been shown to acutely affect the integrity of the blood–brain barrier and blood-retinal barrier and promote the influx of bone-marrow-derived cells [49], [50], [51], [52], while under normal conditions, bone marrow-derived myeloid cells rarely infiltrate the CNS [53]. Consistent with this, it has been clearly demonstrated in the retina that irradiation triggers recruitment of blood-derived monocytes, as detected by transplantation of GFP-labeled bone marrow cells into mice following whole body irradiation [51], [52], [54]. This influx of GFP+ cells into the retina was prevented by shielding of the head, demonstrating that irradiation of the retina is required and that normal microglial cell turnover from circulating monocytes is low [52], [55].

In contrast, Howell et al. report that irradiation of the head or eyes of the D2 mouse at 2 months of age prevents proinflammatory monocytes from entering the optic nerve when analyzed at 10.5 months of age, but prior to detectable neuronal damage, and they provide evidence that these infiltrating monocytes contribute to neuronal decline in D2 glaucoma [26]. Thus, monocyte infiltration may be a critical component of disease in this model. However, it remains to be determined how early irradiation inhibits the invasion of monocytes at much later ages, especially when in other models irradiation promotes monocyte invasion [52]. Importantly, bone marrow-derived myeloid cells may have functions or responses that are distinct from those of resident microglia [53], [56], [57], potentially impacting the course of disease. In our study, Iba1 does not distinguish macrophages from microglia, so it is possible that macrophages are recruited at early stages as well. It remains to be determined whether this contributes to the effects of irradiation in the D2 model of glaucoma.

## Supporting Information

Figure S1 **Experimental design.** *(A)* D2 mice received a single high-dose of X-irradiation targeting their eyes and visual pathways at a prepathological age of 4 to 6 weeks of age. Microglial changes following irradiation were examined in a cohort of young mice, while optic neuropathy was measured in aged mice. Identical analysis was performed in age-matched non-irradiated D2 mice as well as non-glaucoma D2G mice. *(B)* Radiation was confined to the rostral half of the head targeting eyes and visual pathways (orange rectangle). This area was consistently positioned with reference collimated light (white rectangle) and focusing LEDs. The nose and regions behind the ears (5-mm post-lambda, **λ**) were spared, as was the body by shielding with lead plates.
(TIF)

Figure S2 **Intraocular pressure (IOP) elevated with similar patterns in D2 mice from 2 to 8 months of age, regardless of irradiation.** A summary of mean IOP per month (and sample size) shows that irradiation did not prevent IOP elevation.
(TIF)

Figure S3 **Irradiation selectively induces apoptosis in retinal microglia.** Representative retinal wholemounts from non-irradiated and irradiated D2.CX3CR1^gfp/+^ mice, where microglia expresses GFP under the control of fractalkine-receptor locus (optic disc position indicated by asterisk). Apoptotic cells were detected with SR-FLIVO, which selectively binds cleaved caspases and emits at 600 nm. *(A)* Non-irradiated retinas show only very small apoptotic cells positioned along large blood vessels, probably corresponding to pericytes. *(B)* Irradiated retinas, instead, show numerous and conspicuous apoptotic microglia mostly localized to the optic disc and central retina. Some microglial cells are detectable in the mid-peripheral retina (arrowheads). Notice that, relative to non-irradiated mice, the regularity of microglial cell tiling is lost, and fewer cells with complex branching are present in the central retina. *(C, D)* Detailed view of the optic disc area (inset in A, B) showing the overlay of FLIVO and GFP highlights the virtual absence of apoptosis in the non-irradiated retina, and the clear overlap of both stainings to cells with activated shape. n = 3 per group. Scale bars, 100 µm *(A, B)*, 10 µm *(C, D)*.
(TIF)

Figure S4 **Irradiation does not induce early gliosis.** *(A, B)* Images of representative radial sections through the central retina and proximal optic nerve from 3 month-old D2 mice detects comparable GFAP expression in astrocytes, but not in Mueller cells, regardless of treatment. Co-immunostaining for Iba1 shows the relative reduction of microglial cells in the irradiated ONH and OL, relative to the non-irradiated sample. *(C, D)* High-magnification view of astrocytes localized to the OL (insets in A, B) show similar cell size and shape under both experimental conditions, while microglia display morphological signs of activation in the non-irradiated tissue. Scale bar, 100 µm (A, B), 10 µm (C, D).
(TIF)
