# Supplementary figures and images for: Early Reduction of Microglia Activation by Irradiation in a Model of Chronic Glaucoma
Source: PLoS One. 2012 Aug 30;7(8):e43602. doi: 10.1371/journal.pone.0043602 (PMC3431380; doi:10.1371/journal.pone.0043602)

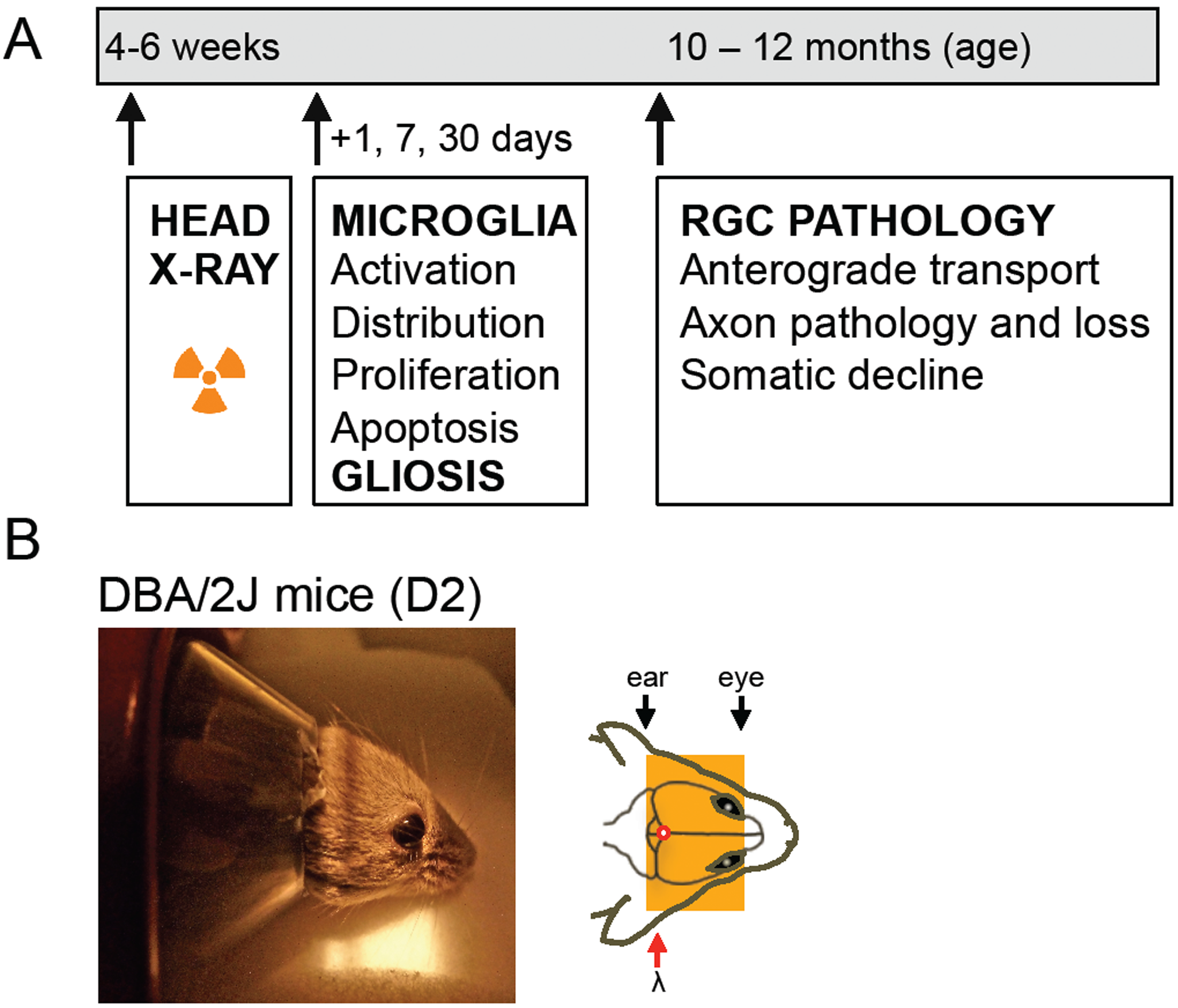

Supplement: Figure S1 — Experimental design. (A) D2 mice received a single high-dose of X-irradiation targeting their eyes and visual pathways at a prepathological age of 4 to 6 weeks of age. Microglial changes following irradiation were examined in a cohort of young mice, while optic neuropathy was measured in aged mice. Identical analysis was performed in age-matched non-irradiated D2 mice as well as non-glaucoma D2G mice. (B) Radiation was confined to the rostral half of the head targeting eyes and visual pathways (orange rectangle). This area was consistently positioned with reference collimated light (white rectangle) and focusing LEDs. The nose and regions behind the ears (5-mm post-lambda, λ) were spared, as was the body by shielding with lead plates. (TIF) [file pone.0043602.s001.tif]

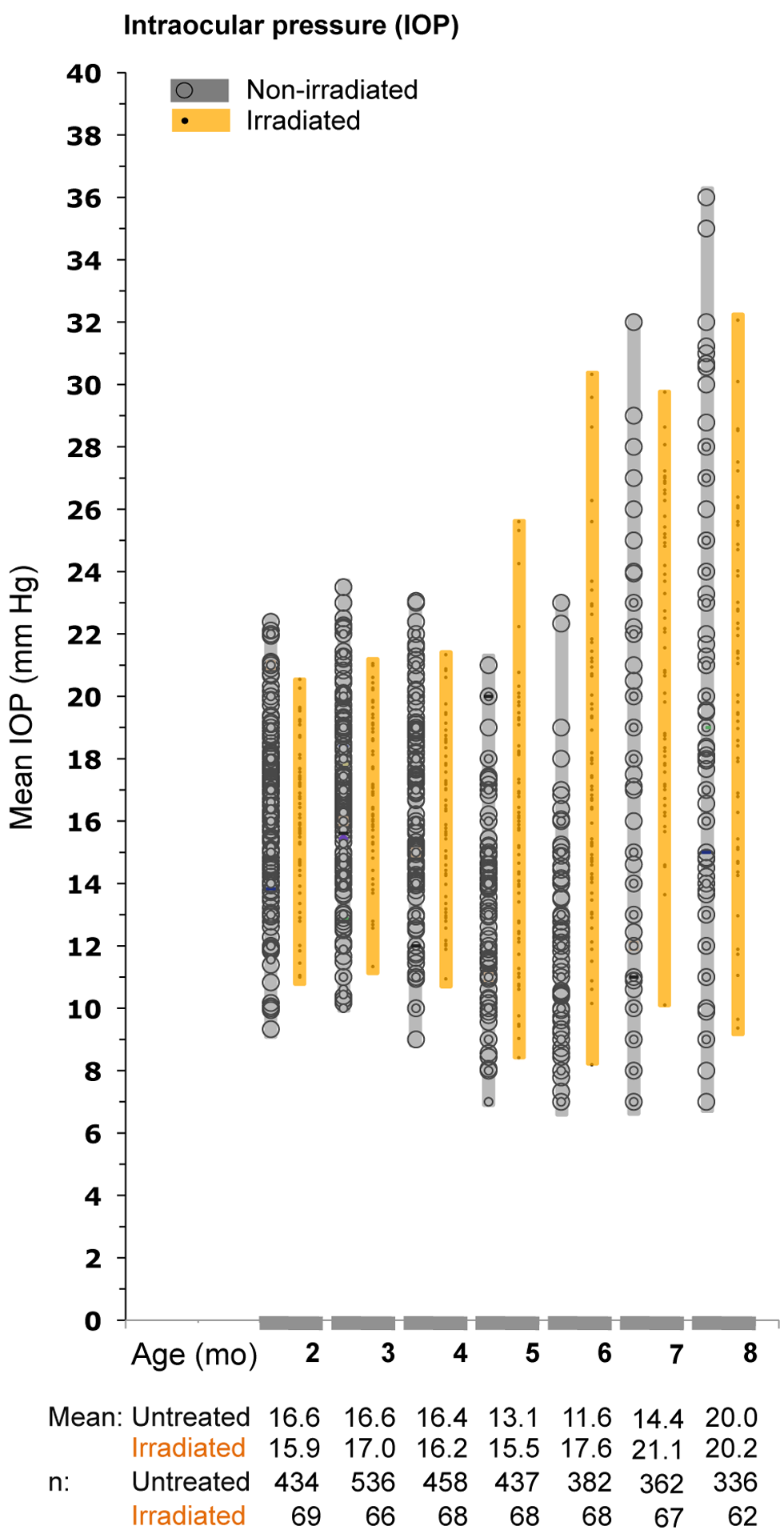

Supplement: Figure S2 — Intraocular pressure (IOP) elevated with similar patterns in D2 mice from 2 to 8 months of age, regardless of irradiation. A summary of mean IOP per month (and sample size) shows that irradiation did not prevent IOP elevation. (TIF) [file pone.0043602.s002.tif]

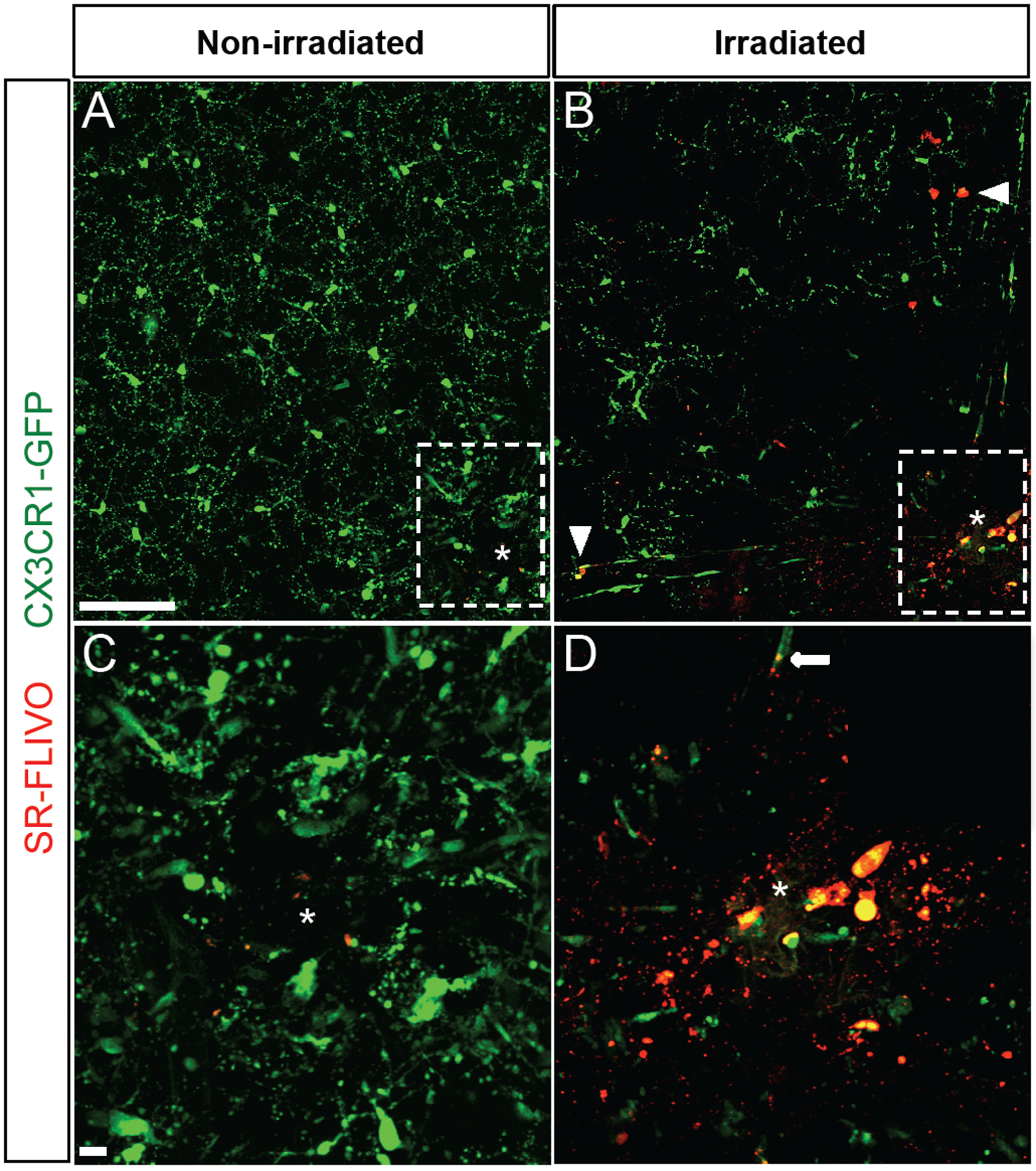

Supplement: Figure S3 — Irradiation selectively induces apoptosis in retinal microglia. Representative retinal wholemounts from non-irradiated and irradiated D2.CX3CR1gfp/+ mice, where microglia expresses GFP under the control of fractalkine-receptor locus (optic disc position indicated by asterisk). Apoptotic cells were detected with SR-FLIVO, which selectively binds cleaved caspases and emits at 600 nm. (A) Non-irradiated retinas show only very small apoptotic cells positioned along large blood vessels, probably corresponding to pericytes. (B) Irradiated retinas, instead, show numerous and conspicuous apoptotic microglia mostly localized to the optic disc and central retina. Some microglial cells are detectable in the mid-peripheral retina (arrowheads). Notice that, relative to non-irradiated mice, the regularity of microglial cell tiling is lost, and fewer cells with complex branching are present in the central retina. (C, D) Detailed view of the optic disc area (inset in A, B) showing the overlay of FLIVO and GFP highlights the virtual absence of apoptosis in the non-irradiated retina, and the clear overlap of both stainings to cells with activated shape. n = 3 per group. Scale bars, 100 µm (A, B), 10 µm (C, D). (TIF) [file pone.0043602.s003.tif]

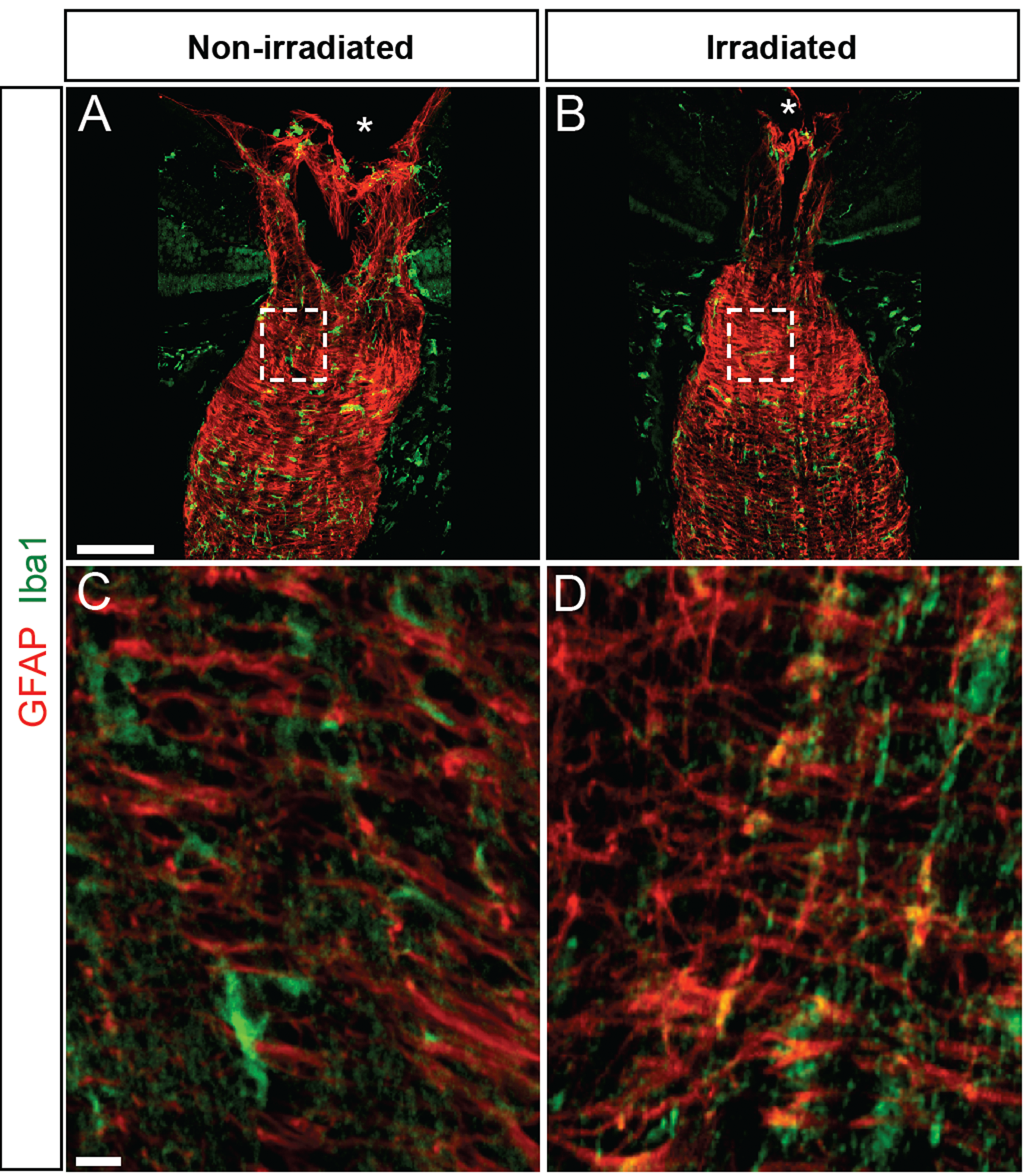

Supplement: Figure S4 — Irradiation does not induce early gliosis. (A, B) Images of representative radial sections through the central retina and proximal optic nerve from 3 month-old D2 mice detects comparable GFAP expression in astrocytes, but not in Mueller cells, regardless of treatment. Co-immunostaining for Iba1 shows the relative reduction of microglial cells in the irradiated ONH and OL, relative to the non-irradiated sample. (C, D) High-magnification view of astrocytes localized to the OL (insets in A, B) show similar cell size and shape under both experimental conditions, while microglia display morphological signs of activation in the non-irradiated tissue. Scale bar, 100 µm (A, B), 10 µm (C, D). (TIF) [file pone.0043602.s004.tif]
